# Results of field testing of municipal solid waste by combination of CPTU and MASW

**DOI:** 10.1016/j.dib.2018.05.109

**Published:** 2018-05-24

**Authors:** Vadim Ofrikhter, Ian Ofrikhter, Mikhail Bezgodov

**Affiliations:** Chair “Construction operations and geotechnics”, Faculty of civil engineering, Perm National Research Polytechnic University, 29, Komsomolsky av., Perm 614990, Russian Federation

## Abstract

The cone penetration testing of MSW by piezocone (CPTU) in combination with the multichannel analysis of surface waves (MASW) provide extensive data about estimated values of physico-mechanical properties of wastes, which are usually unavailable in traditional approaches and which can be directly used in geotechnical stability calculations of the waste massif

**Specifications Table**

**Table:** 

Subject area	*Civil Engineering*
More specific subject area	*Environmental Geotechnics*
Type of data	*Figures*
How data was acquired	*Field CPT(U) testing by cone (piezocone), field survey by multichannel analysis of surface waves (MASW)*
Data format	*Raw, filtered*
Experimental factors	Aged MSW mass (3–7 years old) was tested. No special measures were taken before the testing
Experimental features	*Waste profile was determined by filtered results of CPTU method as well as measured results of MASW method, waste properties were estimated by combination of CPTU and MASW, profile of the specific gravity along the depth of the solid waste massif has been plotted*
Data source location	Perm region, Russian Federation
Data accessibility	The data is available within this article

**Value of the data**

• The data presents the new approach to get field results of municipal solid waste testing by combination of small-invasive (CPTU) and non-destructive (MASW) methods.
• Obtained data were used for instant assessment of municipal solid waste as soil-like material as well as waste profile and specific gravity profile.
• Combination of small-invasive (CPTU) and non-destructive (MASW) methods could be used by researchers as non-expensive approach for instant preliminary estimation of municipal solid waste properties and waste profile.

## 1 Data

**Fig. 1:**
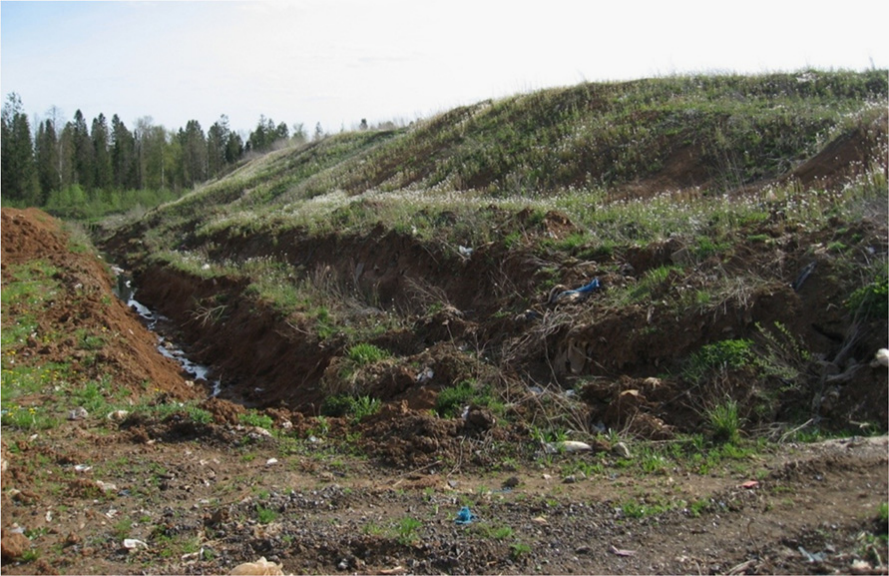
General view of waste pile.

**Fig. 2:**
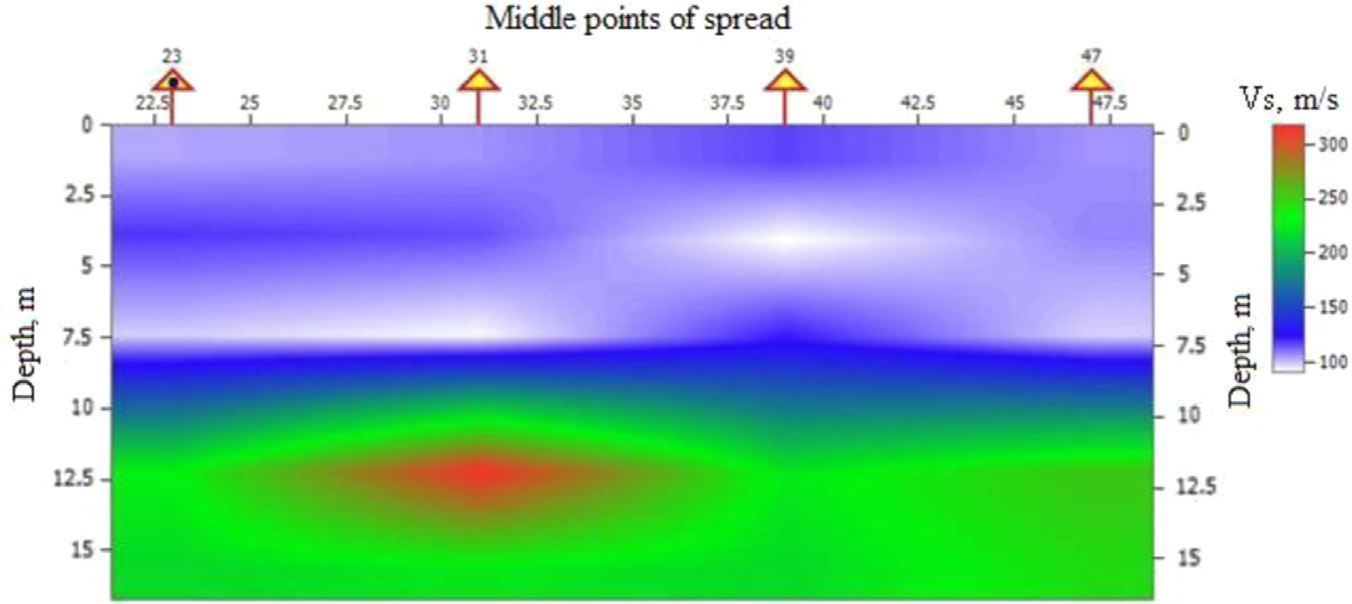
Profile of shear-wave velocity between middle points of spread.

**Fig. 3:**
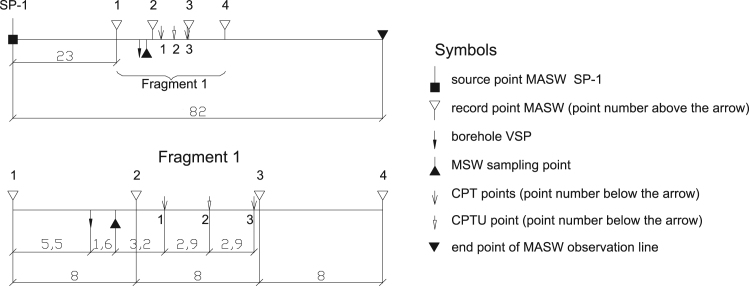
Connection of the boreholes and penetration points to the MASW observation line. Length of MASW observation line is 82 м. All dimensions are in m.

**Fig. 4:**
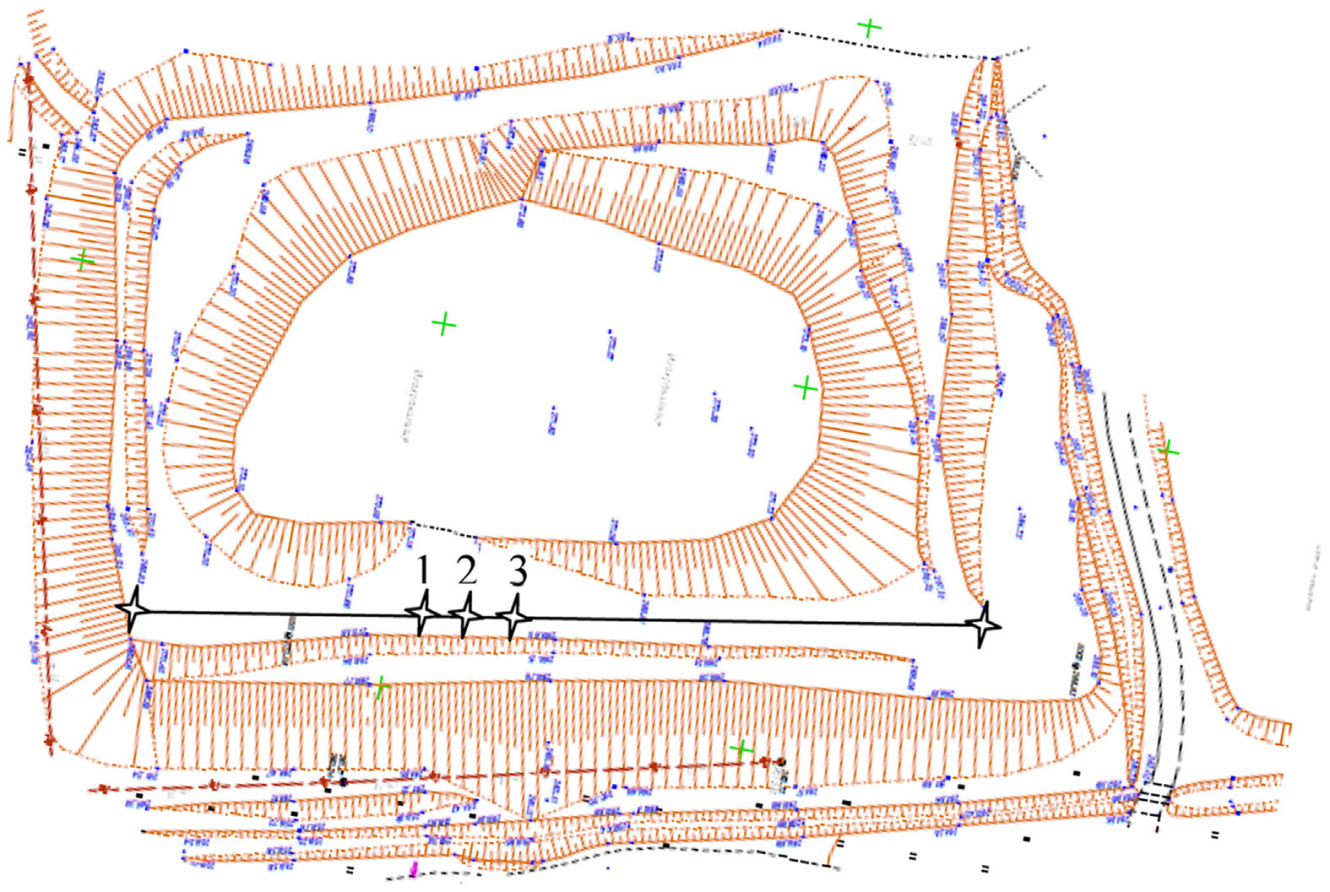
Location of CPT(U) sounding points on the MASW observation line (- MASW observation line); 1, 3 – CPT points; 2 – CPTU point.

**Fig. 5:**
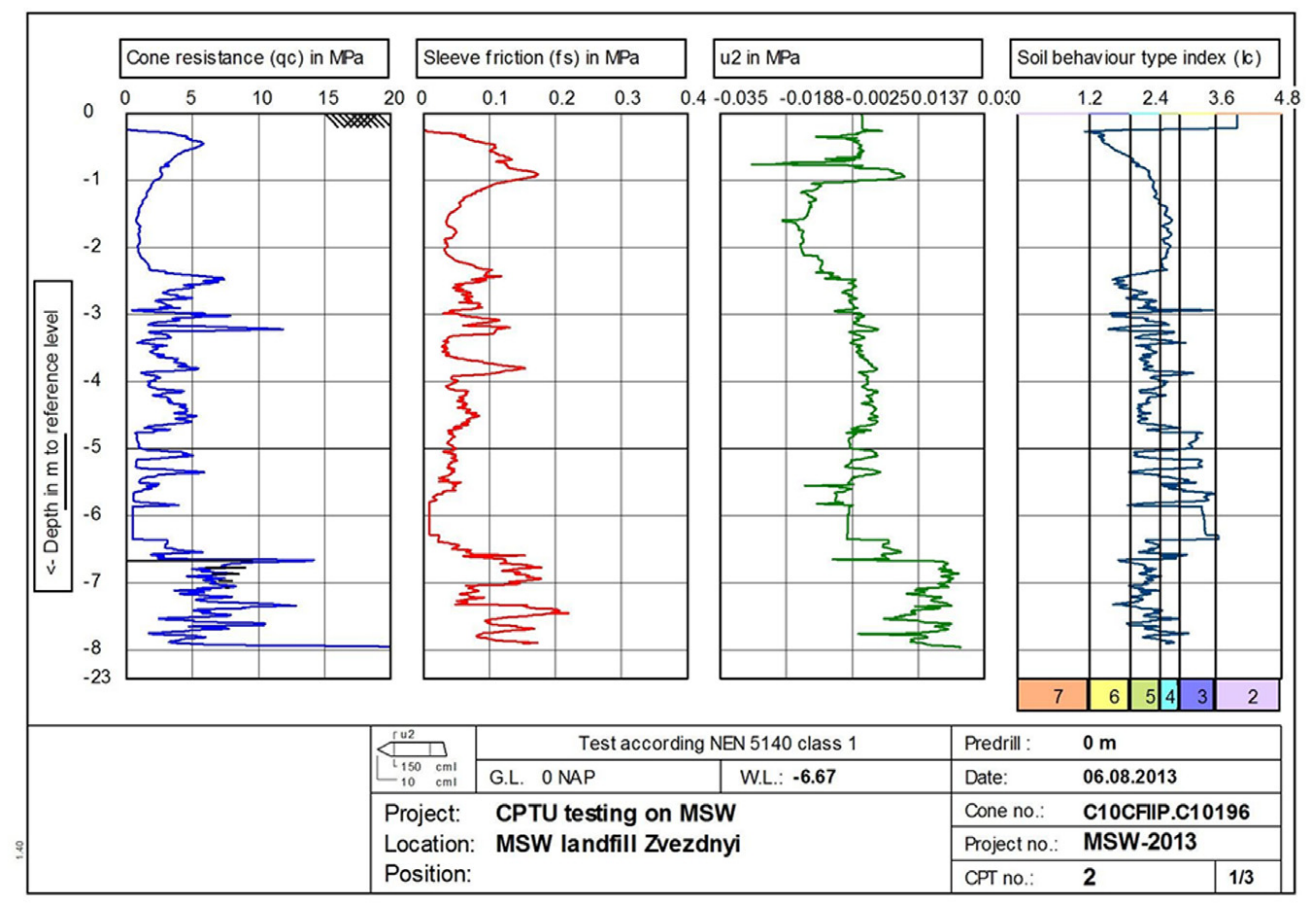
CPTU raw results at point 2. Results in columns from left to right: 1 – cone resistance (*q_c_*), MPa; 2 – sleeve friction (*f_s_*), MPa; 3 – pore pressure (*u*_2_); 4 – soil behavior type index according to Robertson classification (*I*_*c*_) [4]. Types of soil behavior *I*_*c*_: 2 – organic soils, 3 – clay, 4 – silt mixture, 5 – sand mixture, 6 – sand clean to silty, 7 – gravelly sand. Vertical scale – penetration depth in m.

**Fig. 6:**
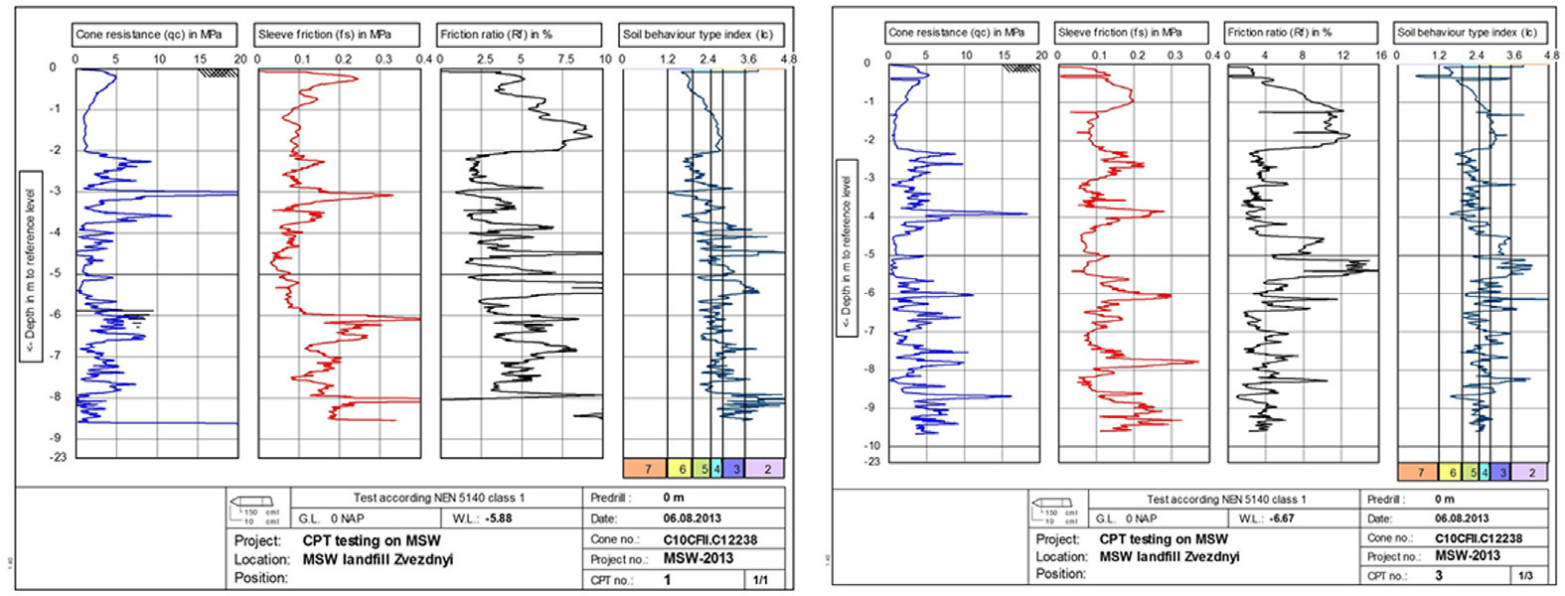
CPT raw results at point 1 (left) and point 3 (right). Results in columns from left to right: 1 – cone resistance (*q_c_*), MPa; 2 – sleeve friction (*f_s_*), MPa; 3 – friction ratio (Rf), %; 4 – soil behavior type index according to Robertson classification (*I*_*c*_) [4]. Types of soil behavior *I*_*c*_: 2 – organic soils, 3 – clay, 4 – silt mixture, 5 – sand mixture, 6 – sand clean to silty, 7 – gravelly sand. Vertical scale – penetration depth in m.

**Fig. 7:**
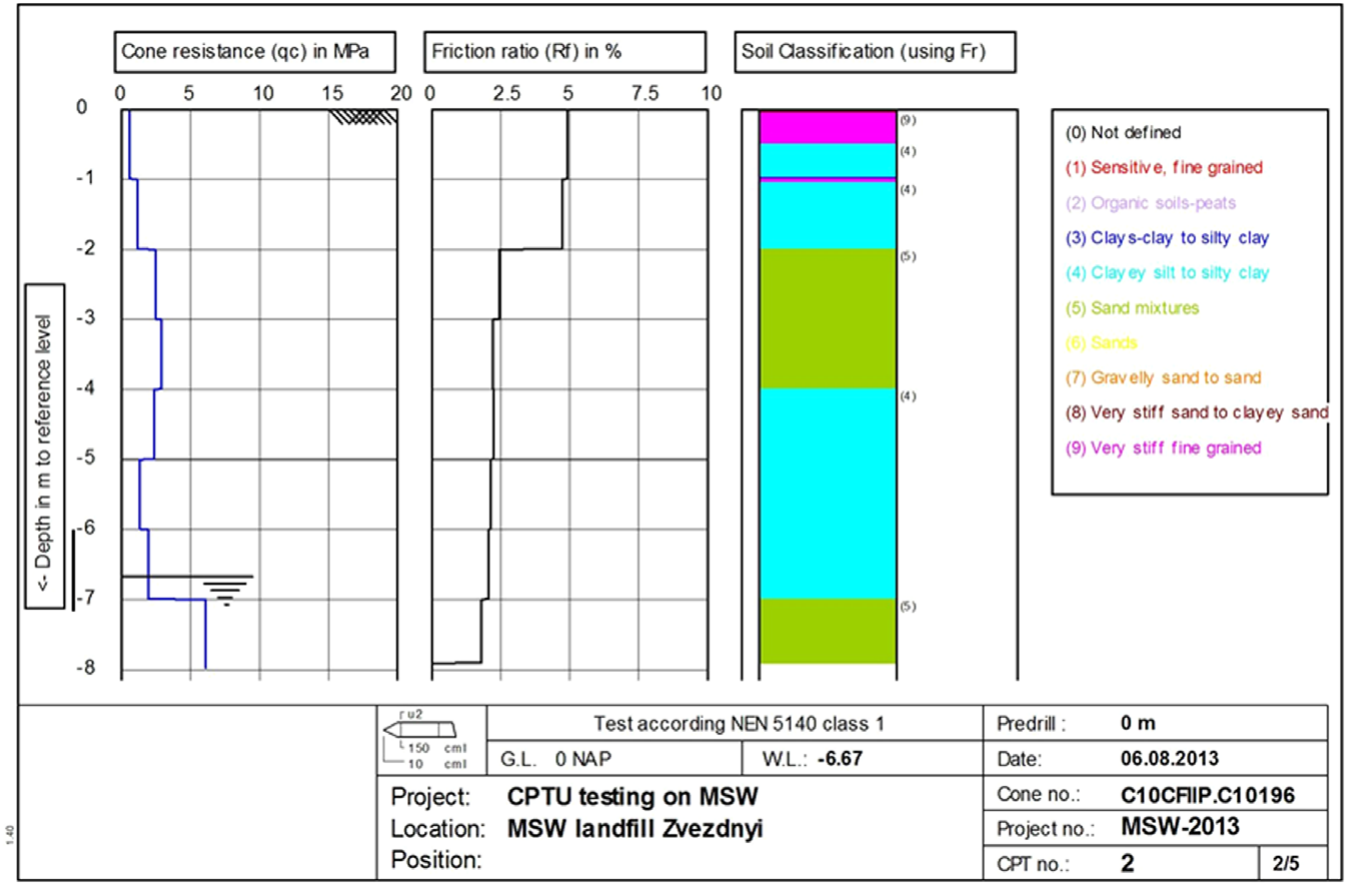
«Filtered» mean results of CPTU testing at point 2. CPTU results in columns from left to right: cone resistance (*q*_c_), friction ratio (*R_f_*), soil type according to Robertson classification [4] (*I*_c_). Vertical scale – penetration depth in m.

**Fig. 8:**
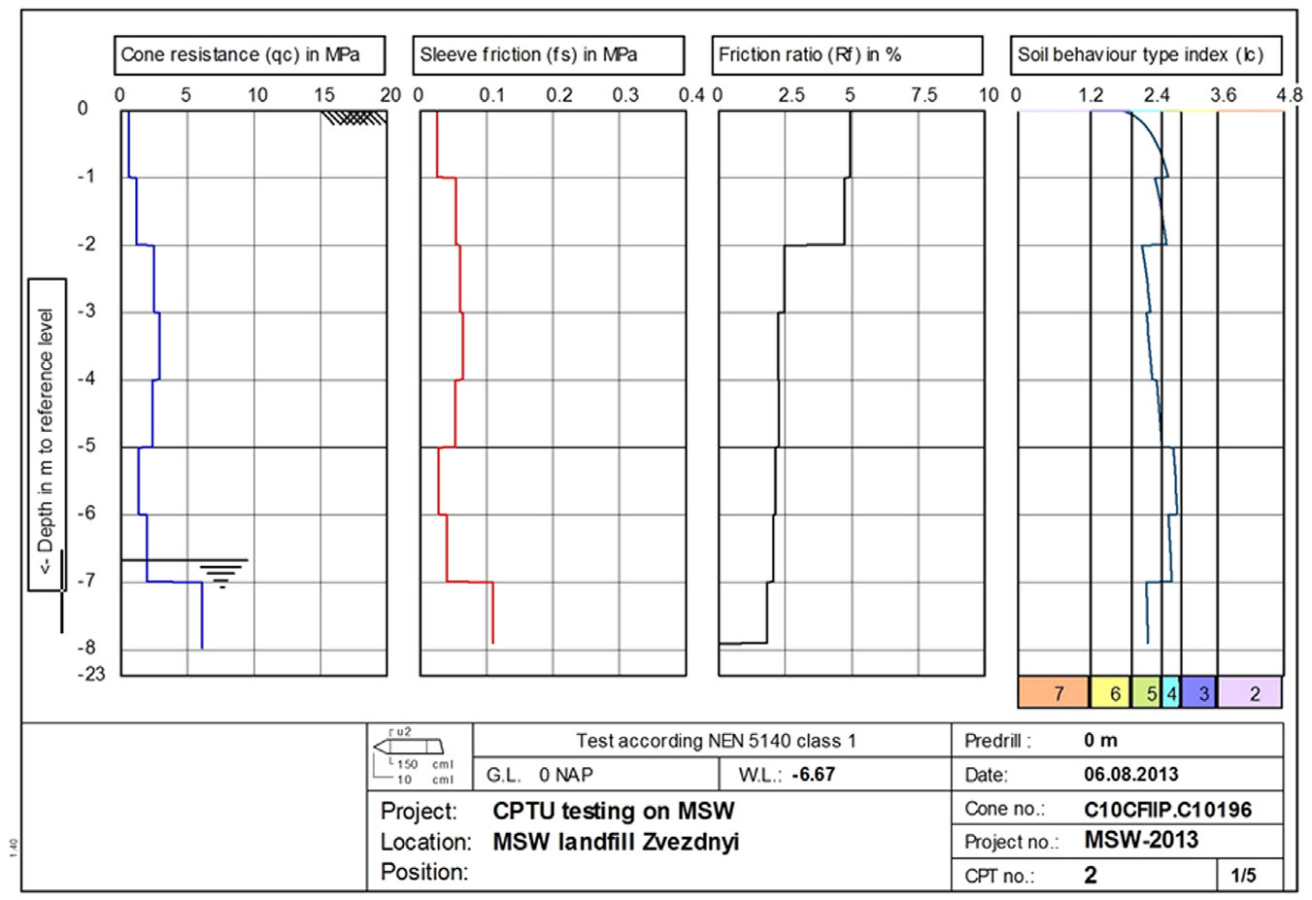
Mean geometrical values of CPTU results at point 2. CPTU results in columns from left to right: cone resistance (*q*_*c*_), sleeve friction (*f*_*s*_), friction ratio (Rf), soil behaviour type index according to Robertson classification (*I*_*c*_) [4]. Types of soil behavior *I*_*c*_: 2 – organic soils, 3 – clay, 4 – silt mixture, 5 – sand mixture, 6 – sand clean to silty, 7 – gravelly sand.

**Fig. 9:**
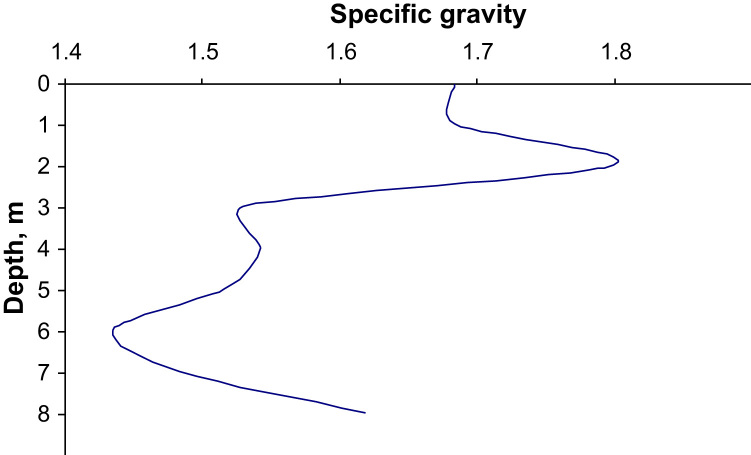
MSW specific gravity profile based on CPTU results.

The dataset of the paper provides information about results of MASW survey and CPTU testing of municipal solid waste massif. Fig. 1, Fig. 3, Fig. 4 show general view location and connection of testing points. Fig. 5, Fig. 6 present raw results of CPT and CPTU testing on three sounding points. Profile of shear wave velocity is shown on Fig. 2. Filtered results of CPTU sounding as well as soil types are shown in Fig. 7, Fig. 8. The profile of the estimated values of the MSW specific gravity according to the results of CPTU testing by the method [1], [2] is shown in Fig. 9.

## 2 Experimental design, materials and methods

At the first stage registration of surface waves was done by a telemetric 24-channel seismic exploration system.

Investigation of a waste pile profile was made on a landfill area of 115 m×145 m in size and around 7.5 m in height on top of the aged MSW mass (3–7 years old) (Fig. 1).

The subsoil is represented by the low-plastic clay layer of 1.7 m in thickness, which is underlaid by the silty low-plastic loamy clay layer of 3.4 m in thickness. Two-dimensional in-line offset end-on spread geometry for the surface waves registration was applied. After the survey and data record completion at each shot point, all the system, including the 24-channel receiver spread and the shot point, was carried to the distance of 8 m.

After processing and material interpretation the two-dimensional profile of shear-wave velocities was plotted by the interpolation between the obtained vertical profiles (Fig. 2)

In Fig. 2 there are three clearly visible layers, the top of which is the mass of waste. The upper layer of the landfill base consisting of low-plastic clay is located under the waste, and the third layer is silty low-plastic loamy clay. The average velocity of shear waves in the first layer from the surface (waste) was 110 m/s, in the second (clay) – 150 m/s and in the third (loamy clay) – 260 m/s.

The method of the multichannel analysis of surface waves (MASW) is the most inexpensive and fast way of field testing of soils and soil-like materials, the selection and restoration of undisturbed samples of which are impossible. MSW attributes to these types of material. The outcome of the research is a wave velocity profile.

A total of 3 tests were performed, including 2 CPT tests and one CPTU test. The connection of the sounding points to the MASW survey line [3] is shown in Fig. 3. The position of sounding points is shown in Fig. 4.

The results of CPT tests allow us to identify MSW as a soil-like material and to obtain information on the structure of waste pile and underlying soil, the type of soil behavior and the mechanical characteristics of the waste. Solid inclusions appear as local peaks on the sounding charts, which quickly disappear as the cone tip passes through the inclusions (Fig. 5).

Based on the results of the sounding, it is possible to distinguish two characteristic layers - the cover soil and solid waste. The transition from the cover layer of soil, consisting of loam, to the layer of solid waste is clearly recorded on the sounding charts at all three points at a depth of 2–2.5 m (Fig. 5, Fig. 6). Submergence of the probe below the groundwater level led to an increase in pore pressure during the CPTU test at point 2 (Fig. 5).

Due to the fact that the results of the CPT tests of MSW are characterized by a large variation of the readings, they were filtered by the mean geometric method [5], and then graphically represented by geometric mean values at every meter in depth. The "filtered" average values of test results are shown in Fig. 7.

The results of CPT tests at the landfill site correspond to typical values for clays, clayey mixtures and sand mixtures (see Fig. 7, Fig. 8).

The profile of the estimated values of the MSW specific gravity according to the results of CPTU testing by the method [1], [2] is shown in Fig. 9.
