# Supplementary material for: Results of field testing of municipal solid waste by combination of CPTU and MASW
Source: Data Brief. 2018 May 24;19:883–9. doi: 10.1016/j.dib.2018.05.109 (PMC5997941; doi:10.1016/j.dib.2018.05.109)
Supplement: Supplementary file 1 — Supplementary material [file mmc1.docx]

Conflict of interests

There is no conflict of interests.
